# Depth-driven decline in viral diversity unveils potential novel viruses in global deep-sea ecosystems

**DOI:** 10.1099/mic.0.001632

**Published:** 2025-12-10

**Authors:** Melany Calderón-Osorno, Keilor Rojas-Jimenez

**Affiliations:** 1 Costa Rica National High Technology Center (CeNAT), Pavas, 10108, San José, Costa Rica; 2 Maestría académica en Biología con enfásis en genética y biología molecular, University of Costa Rica, San Pedro, 11501-2060, San José, Costa Rica; 3 Biology School, University of Costa Rica, 11501-2060, San José, Costa Rica

**Keywords:** deep sea, metagenomics, virome, viromics

## Abstract

Deep-sea ecosystems remain poorly understood due to exploration challenges. Despite the advancements metagenomics have brought to the understanding of the ocean microbiome, the diversity of marine viruses, particularly in the deep sea, is still not well characterized. In this study, we analysed the impact of depth on the composition and diversity of marine viruses in deep-sea waters at a global scale. Raw reads from deep-sea shotgun DNA sequences were retrieved from the Tara and Malaspina expeditions, encompassing depths from 270 to 4,005 m. A total of 80 samples containing viral reads were identified and analysed through a comprehensive bioinformatics pipeline, including quality assessment, taxonomic classification and metabolic annotation. The analysis reveals that microbial viral diversity significantly decreases with depth, with shallower waters exhibiting higher species richness. We determined that a substantial proportion of deep-sea viral sequences remains unclassified – up to 31.9% at depths of 270–1,000 m and 9.6% at 2,400–4,005 m. Additionally, a higher abundance of auxiliary metabolic genes was observed at shallower depths, indicating potential roles in host metabolism and adaptation. Our findings reveal the deep ocean as a vast, largely unexplored source of microbial viral diversity. This research emphasizes how depth influences viral diversity and community makeup in deep-sea environments, underscoring the need for further exploration to fully grasp their complexity and ecological roles.

## Data Summary

The data and pipeline needed to reproduce this work are available at GitLab (https://gitlab.com/CNCA_CeNAT/deep-sea-virome).

## Introduction

Deep-sea ecosystems cover ~65% of the Earth’s surface and play crucial roles in biomass production and biogeochemical cycles [1,2]. However, the challenges of deep-sea exploration make it one of the least understood ecological regions on Earth [3]. The deep sea, defined as environments beyond 200 m, encompasses many of Earth’s extreme conditions. It has an average depth of ~4.2 km, with temperatures below 4 °C and a hydrostatic pressure of 400 atmospheres. The absence of sunlight prevents photosynthetic primary production below 200 m, further challenging the functioning of deep-sea systems. Additionally, the deep sea includes Earth’s largest hypoxic and anoxic environments [4].

The limited exploration and scientific research, due to the remote and harsh conditions of the deep ocean, have resulted in a partial understanding of this vast ecosystem [5]. While metagenomics has significantly advanced our knowledge of the ocean microbiome by revealing its extensive phylogenetic and metabolic diversity [6,7], our understanding of marine viruses – along with their diversity and variability – remains incomplete compared to bacterial communities. This gap is largely due to the absence of universal gene markers for studying viral communities and the relatively recent development and application of culture-independent high-throughput sequencing methods [7].

Viruses are the most abundant entities across all habitats and serve as a major reservoir of genetic diversity. Most environmental viruses are bacteriophages, which infect bacteria [8]. Due to their vast abundance and genetic diversity, viruses play crucial roles in marine ecosystems. They regulate host populations and influence community structure by killing their hosts. Additionally, viruses impact host diversity, evolution and environmental adaptation through mechanisms such as horizontal gene transfer, resistance selection and modulation of host metabolism [9]. They also drive biogeochemical cycles by releasing intracellular organic matter from their hosts and facilitating the transformation of particulate organic matter into dissolved organic matter. Furthermore, viruses contribute to microbial-mediated biogeochemical processes by expressing auxiliary metabolic genes (AMGs) [10]. Marine viruses often contain host-derived metabolic genes, AMGs, which are hypothesized to increase viral replication by enhancing key steps in host metabolism [11].

In recent years, several marine and ocean viromes have been identified and characterized through high-throughput sequencing [3,7, 10]. Over the past two decades, viral communities from various marine environments have been increasingly resolved through metagenomics. This progress has been driven by major expeditions and datasets, including the Global Ocean Sampling Expedition [12], Pacific Ocean Virome [13], Tara Ocean Expedition [14], Malaspina Expedition [15,16] and Tara Oceans Polar Circle expedition [7,17].

Although some deep-sea viruses have been characterized, the viral communities across global deep-sea environments have not been extensively explored. To our knowledge, only a few published studies have explored the global diversity and distribution of deep-sea viruses [3,18]. However, these studies do not examine how viral populations vary along depth gradients. Additionally, AMGs have not been studied in the context of depth gradients. Therefore, we also aimed to determine if deep-sea viruses contain AMGs and how these genes are affected by depth gradients.

In this study, we characterize the virome (virus population) of deep-sea metagenomes through taxonomic and functional annotation. Our analysis focuses on viromes derived from 80 metagenomes collected from two distinct depth groups. We utilize a bioinformatics pipeline that includes tools for extracting virus sequences, taxonomic annotation and pathway annotation.

## Methods

### Data acquisition

To analyse the global deep-sea virome, we searched for raw reads from deep-sea shotgun sequencing samples of seawater from around the world in the European Bioinformatics Institute European Nucleotide Archive database [19] and NCBI databases [20]. We selected two main expedition bioprojects for our study: the Malaspina Expedition 2010 (accession number PRJEB40454) [16] and the Tara Oceans Expedition 2009–2013 (accession number PRJEB1787) [17]. The Tara Oceans expedition provided samples from depths of 270 to 1,000 m (*n*=53), while the Malaspina expedition covered depths from 2,400 to 4,005 m (*n*=28). This dataset has not been previously analysed in studies such as [21,22] (Table S1, available in the online Supplementary Material). We retrieved a total of 81 whole-metagenome samples from the Indian Ocean (*n*=18), Atlantic Ocean (*n*=29) and Pacific Ocean (*n*=34) (accession date: 27 September 2023). The sample accession numbers, locations and depths are detailed in Table S2.

### Data processing and profiling

The quality of raw reads was assessed using FastQC v0.11.9 [23], and sequences were subsequently trimmed with fastp v0.20.1 [24], applying a minimum average quality score threshold of 25. To remove human contamination, reads were aligned to the GRCh37 (hg19) reference genome using Bowtie2 v2.5.0 [25].

We employed two complementary strategies for virome characterization: (i) direct profiling of raw reads and (ii) analysis of assembled contigs from all reads. For read-based profiling, the filtered reads were taxonomically classified at the species level using Kraken2 v2.1.2 [26] and Bracken v2.7 [27], with the Kraken2-Bracken Viral reference database (dated 28 December 2024) [28]. The classification results were merged using the *combine_mpa.py* script from KrakenTools [29]. The final merged table was then converted into a microtable object for downstream analysis using microeco v1.13.0 [30].

### Whole metagenome assembly and identification of viral sequences

For assembly-based characterization, all reads from each sample were assembled using metaSPAdes v3.14.1 [31] with k-mer values of 33, 55, 77 and 99 bp. To maximize viral sequence recovery, the assembled contigs were classified using both VirSorter2 v2.2.3 [32] and VIBRANT v1.2.0 [33], allowing us to evaluate which tool performed better for our dataset.

First, the assembled contigs were analysed with VirSorter2 [32] using the ‘dsDNA phage’, ‘ssDNA’, ‘RNA’ and ‘NCLDV’ classifiers, applying a minimum score threshold of 0.75 and a contig length cutoff of ≥5 kb. Contigs meeting this length criterion and obtaining the highest scores in the ‘dsDNA phage’ and ‘ssDNA’ classifiers were considered putative viral sequences, as described in [18]. This filtering step excluded one sample (SRR3960579), in which no sequences ≥5 kb were classified as ‘dsDNA phage’ or ‘ssDNA’. It is important to note that the methodologies used for extraction, processing and sequencing introduce a bias toward DNA viruses.

The second approach employed VIBRANT v1.2.0 [33], a tool capable of classifying dsDNA, ssDNA and RNA viruses. Contigs >5 kb from the metagenome assemblies were processed using default settings to recover viral sequences. The quality of viral sequences identified by both VirSorter2 v2.2.3 [32] and VIBRANT v1.2.0 [33] was then assessed using CheckV v1.0.3 [34].

To further evaluate virome quality, we used metaQUAST v5.2.1 [35]. Key assembly statistics, including virus contig N50 values, maximum contig size, total contig length and the number of contigs per sample, were used to generate summary tables detailing contig length (bp) and count per sample.

This process revealed that VirSorter2 v2.2.3 [32] identified a larger number of viral contig sequences and produced a higher total assembly output compared to VIBRANT v1.2.0 [33]. Consequently, downstream analyses were performed using viral contigs obtained with VirSorter2 v2.2.3 [32].

### Taxonomic assignments and abundance profiles of viruses

ORFs were predicted from viral contig sequences using Prodigal v2.6.2 [36] with the parameters *-p meta -g 11 -m -c*. The resulting ORFs were mapped to the NCBI viral_refseq database (8 July 2024) using CAT v5.3 [37], which utilizes the last common ancestor (LCA) algorithm to assign taxonomic classifications. CAT was chosen over other viral taxonomy tools due to its proven performance with both short and long contigs [38]. To estimate relative abundances within each sample, quality-filtered reads were aligned to the viral contig sequences using Bowtie2 v2.5.0 [25], and read counts were calculated with Pysam v0.22.0 [39].

### Metabolism annotation

VirSorter2 v2.2.3 [32] contigs were subsequently reprocessed using the *prep-for-dramv* flag to prepare for AMG identification with DRAMv [40]. The remaining ‘potential virus’ contigs were clustered into virus populations if they shared 95% average nucleotide identity across 80% of the genome as suggested previously [17] using MMSEQS2 v17 (easy-cluster) [41].

To systematically identify AMGs, we followed the established protocol [17]. Briefly, all viral contigs were annotated using DRAM-v (v1.0.6) [40] with a minimum bit score of 60. A gene was considered a candidate AMG if it was assigned to a metabolic module and/or previously reported as an AMG and had an auxiliary score (indicating the confidence of viral origin) of ≤3. This approach resulted in the construction of a permissive AMG catalogue [17].

We identified permissive AMGs within viral sequences using DRAM-v (v1.0.6) [40] and subsequently assessed the final AMGs using the anvi-estimate-metabolism tool from Anvi'o v8.0 [42]. BAM files obtained from Bowtie2 v2.5.0 [25] were imported for this analysis. AMGs were annotated by matching hits to the KOfam database of functional orthologues and assigning genes to their respective metabolic pathways. A metabolic module was classified as complete if at least 75% of the genes within the module were present.

### Statistical analysis

Data exploration and statistical analyses were performed in the R v4.2.2 environment [43] using various R packages, including microeco v1.13.0 [30], Vegan v2.6 [44], stats v4.2.2 [45] and DESeq2 v1.38.3 [46]. For viral read profiling, alpha and beta diversity analyses were conducted with microeco v1.13.0 [30]. Samples were rarefied to 2,112 reads, and alpha diversity metrics were calculated using the cal_alphadiv() function, while Bray–Curtis similarities were computed with cal_betadiv() from the same package [30]. Alpha diversity comparisons were performed using Kruskal–Wallis tests, while beta diversity was assessed via permutational multivariate analysis of variance (PERMANOVA). Differentially abundant species were identified using LefSe (alpha=0.01) [30], which applies a combination of non-parametric tests and linear discriminant analysis [47].

For viral contig sequence profiling, alpha diversity metrics were calculated with Vegan v2.6 [44], and Bray–Curtis similarities were computed using the metaMDS() function from the same package. Alpha diversity comparisons were conducted using Kruskal–Wallis tests with the kruskal.test() function from stats v4.2.2 [45], while beta diversity analysis was performed using PERMANOVA with the adonis() function from Vegan v2.6 [44].

Normalization of read counts for viral sequence contig taxonomic designation and metabolic pathway annotation was performed using the DESeq() function, with data transformation handled by the normTransform() function from the DESeq2 v1.38.3 [46] R package. The DESeq2 package performs internal normalization by computing the geometric mean of each gene across all samples and then normalizing the read counts by dividing each gene’s count by its respective mean. All plots were generated with the ggplot2 v3.5.0 [48] R package. The complete analysis pipeline (Fig. S1) utilized Python scripts for constructing feature tables and R scripts for subsequent analysis. Both Python and R scripts are available online at GitLab (https://gitlab.com/CNCA_CeNAT/deep-sea-virome).

## Results

### Viral sequence read diversity decreases with depth

We analysed a total of 80 whole-metagenome samples from the Indian Ocean (*n*=18), Atlantic Ocean (*n*=29) and Pacific Ocean (*n*=33) to explore the diversity of viruses inhabiting the deep sea. Currently, two metagenomic approaches are available for studying environmental viral communities: viromes of isolated ambient viruses and bulk metagenomes containing sequences from diverse origins. Both approaches have significantly expanded our understanding of environmental viruses [11].

Using a bulk metagenomic approach, we first assessed viral diversity by analysing filtered reads without assembly. This analysis revealed that species diversity from viral reads was significantly influenced by depth (Wilcoxon test, padj=3.32e-12). A higher number of species was observed at 270–1,000 m, with an average of 242 species and a mean Shannon index of 4.5, compared to 101 species on average at 2,400–4,005 m, where the mean Shannon index was 4.1 (padj=1.31e-7) (Fig. S2a, b).

To assess the impact of ocean and depth groups on viral community composition, we performed a PERMANOVA on Bray–Curtis dissimilarities. The analysis revealed that depth groups (R²=0.16) significantly contributed to the dissimilarity in viral communities (*P*-value=0.001). Visualization of Bray–Curtis dissimilarities using non-metric multidimensional scaling (NMDS) showed that samples tended to cluster according to depth groups (Fig. S2c).

Viral communities were predominantly composed of *Caudoviricetes* and *Megaviricetes* classes. At depths of 270–1,000 m, Caudoviricetes represented an average of 80.1%, slightly decreasing to 78.3% at 2,400–4,005 m. Megaviricetes accounted for 11.4% at 270–1,000 m and 12.6% at 2,400–4,005 m (Fig. S2d). The most abundant species at 270–1,000 m was *Sinsheimervirus phiX174* (padj=5.26e-14), whereas at 2,400–4,005 m, *Gihfavirus pelohabitans* and *Menderavirus mendera* were the most prevalent (padj=1.85e-7 and 1.51e-08, respectively) (Fig. S3).

### Viral sequence recovery and assembly metrics decrease with depth

Bioinformatics strategies can vary significantly in their efficiency for recovering viral populations [11]. To identify viral sequences from bulk metagenomes, we employed two pipelines: VirSorter2 v2.2.3 [32] and VIBRANT v1.2.0 [33]. VirSorter2 recovered an average of 95 putative viral sequences per sample (7,574 in total), while VIBRANT identified an average of 86 sequences per sample (6,912 in total). In our dataset, the choice of pipeline did not significantly affect the number of recovered viruses (*P*=0.77), total assembly size (*P*=0.83) or N50 value (*P*=0.95), based on Kruskal–Wallis tests. However, VirSorter2 produced slightly better assembly metrics overall. It yielded a larger average assembly size (972 kbp) compared to VIBRANT (929 kbp). Notably, the largest assembly recovered by VirSorter2 reached 7.1 Mbp, whereas the maximum obtained with VIBRANT was 6.0 Mbp (Fig. S4).

Viral sequences recovered by VirSorter2 v2.2.3 [32] were significantly influenced by depth, with a higher number of sequences identified at 270–1,000 m (average of 137) compared to 2,400–4,005 m (average of 11) (*P*-value=4.7e-10, Wilcoxon test). The total assembly size was also larger at 270–1,000 m (average of 1.4 Mbp) compared to 2,400–4,005 m (average of 136 kbp) (*P*-value=1.3e-9, Wilcoxon test). Additionally, the largest contig size was greater at 270–1,000 m (average of 99 kbp) than at 2,400–4,005 m (average of 40 kbp) (P-value=4.0e-5, Wilcoxon test) (Fig. S5).

**Fig. 1.**
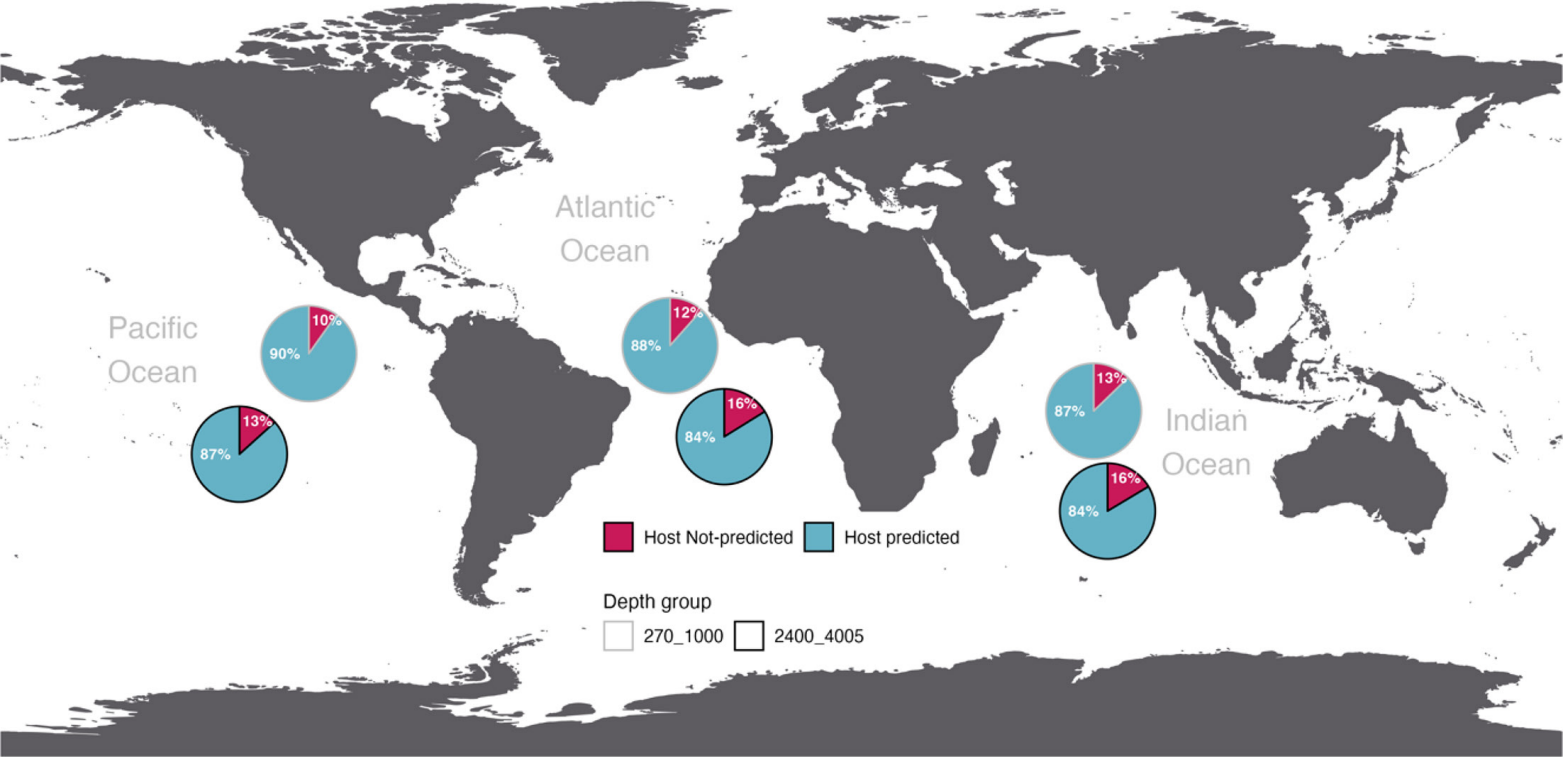
Viral community structure of global deep-sea samples, indicating the proportion of known and unknown viruses. Pie charts with black borders represent samples from depths of 2,400–4,005 m.

According to CheckV analysis, an average of 88.6% of sequences from depths of 270 to 1,000 m were identified as known viruses in the NCBI RefSeq database (host-predicted), while 84.7% were identified at depths of 2,400 to 4,005 m. Host-predicted sequences were most prevalent in the Pacific Ocean at 270–1,000 m, where they accounted for 90.1% of the total. In contrast, the highest proportions of unclassified sequences were found in the Indian and Atlantic Oceans at 2,400–4,005 m, representing 16.4 and 16.2%, respectively (Fig. 1).

Of the viral contigs identified as known viruses by VirSorter2 v2.2.3 [32] (86% across both depth groups), only 65% were detected by VIBRANT v1.2.0 [33]. The remaining contigs were not recovered by VIBRANT v1.2.0 (Fig. S6).

### Viruses in deep water are novel

Viral contig sequences that passed quality assessment with CheckV were assigned to species-level taxonomy. Taxonomic affiliations were determined by comparing predicted ORFs against the NCBI viral_RefSeq database (8 July 2024) using the LCA algorithm. Species diversity was significantly influenced by depth (Wilcoxon test, *P*=8.6e-10), with a greater number of species detected at depths of 270–1,000 m (mean=34 species) compared to 2,400–4,005 m (mean=5 species) (Fig. S7a). Shallower depths also showed greater evenness and overall diversity, with a mean Shannon index of 1.6, whereas deeper samples exhibited a lower mean index of 0.9 (*P*=3e-6) (Fig. S7b).

A PERMANOVA on Bray–Curtis dissimilarities assessed the impact of ocean and depth groups on viral community composition. The analysis revealed that both depth (R²=0.12) and ocean (R²=0.08) significantly contributed to the dissimilarity in viral communities (*P*-value=0.001). Visualization of Bray–Curtis dissimilarities using the NMDS plot showed that samples tended to cluster according to depth groups (Fig. S7c).

**Fig. 2.**
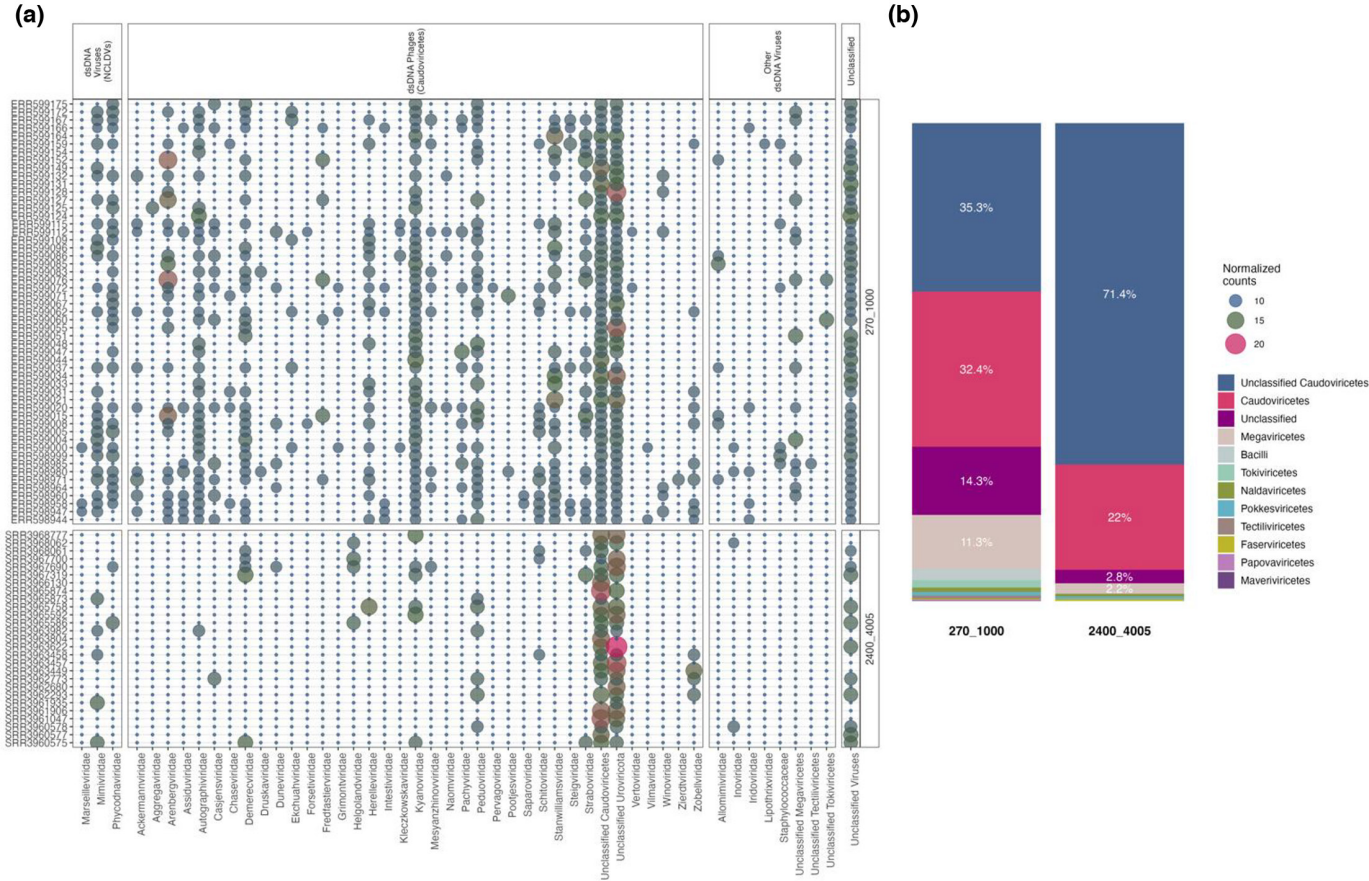
Taxonomic diversity of deep-sea viruses. (**a**) Relative abundance of viruses at the family level. (**b**) Relative abundance at the class level, with the first pie chart corresponding to 270–1,000 m and the second to 2,400–4,005 m.

Although most identified viruses were dsDNA viruses, their sub-population structure varied in relative abundance. Viral abundance was higher at depths of 270–1,000 m (mean: 6.5 normalized counts) compared to 2,400–4,005 m (mean: 6.0 normalized counts). A substantial portion of sequences could not be matched to known viruses in reference databases, with unclassified Caudoviricetes and unclassified *Uroviricota* representing the most dominant groups. Sequences assigned to unclassified Caudoviricetes showed a higher average relative abundance at 2,400–4,005 m (12.6 normalized counts) than at 270–1,000 m (10.1). Similarly, unclassified Uroviricota were more abundant at greater depths (12.2 vs. 10.4 normalized counts). Sequences not assigned to any known viral class had a mean relative abundance of 7.7 normalized counts at 2,400–4,005 m, compared to 9.4 at 270–1,000 m (Fig. 2a).

A significant proportion of sequences were classified as unclassified members of the Caudoviricetes class, accounting for 35.3% of the viral community at 270–1,000 m and increasing to 71.4% at 2,400–4,005 m. Classified Caudoviricetes were the second most dominant group at both depths, comprising 32.4% at 270–1,000 m and 22.0% at 2,400–4,005 m (Fig. 2b). At the species level, unclassified viruses were the most abundant group at 2,400–4,005 m, with an average relative abundance of 31.9%, compared to 9.6% at 270–1,000 m. Among identified species, *Wroclawvirus PA5oct* was the second most abundant at 270–1,000 m (average of 7.0%), while *Eowynvirus eowyn* ranked second at 2,400–4,005 m (average of 5.2%) (Fig. S8).

### Metabolic activity of deep-sea viruses via AMG expression

Viruses can influence host metabolism through the expression of AMGs [11]. To explore the effects of viral AMGs on host metabolic pathways, AMGs were identified from viral sequence contigs using DRAMv [40] and Anvi'o v8.0 [42] and functionally annotated with KEGG databases. After manual curation, a total of 157 genes were identified, which clustered by depth (Fig. S9).

KEGG annotation revealed that genes from deep-sea viruses were involved in various metabolic pathways, with the metabolism of cofactors and vitamins, as well as nucleotide metabolism, being the most abundant in both depth zones. Cofactor and vitamin metabolism was the most enriched pathway at 2,400–4,005 m (averaging 26.7%) and also highly represented at 270–1,000 m (25.9%). In contrast, nucleotide metabolism was most abundant at 270–1,000 m (21.0%), followed closely by its presence at 2,400–4,005 m (19.8%) (Fig. S10).

**Fig. 3.**
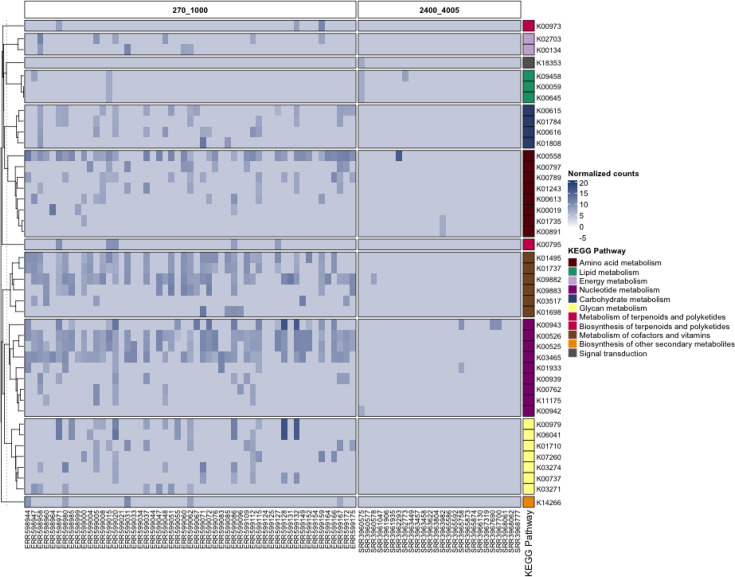
Relative abundance of viral AMGs in deep-sea samples. Kyoto Encyclopedia of Genes and Genomes (KEGG) metabolic categories are coloured according to the legend.

To explore the relative abundance of AMGs, clean reads were mapped to viral sequences carrying AMGs. As shown in Fig. 3, the viromes from 270 to 1,000 m and 2,400–4,005 m exhibited distinct AMG profiles. Generally, there was a significant (padj<0.01) increase in the relative abundance of AMGs related to cofactor and vitamin metabolism, nucleotide metabolism and glycan metabolism. At 270–1,000 m, enzymes associated with these pathways were more abundant. Notably, DNA (cytosine-5)-methyltransferase 1 (K00558, involved in amino acid metabolism) showed the highest relative abundance (7.4 normalized counts), followed by ribonucleoside-diphosphate reductase (K00525 and K00526, involved in nucleotide metabolism) with an average of 7.3 normalized counts and thymidylate synthase (K03465, also involved in nucleotide metabolism) with an average of 7.1 normalized counts (Fig. 3).

## Discussion

Although numerous viruses have been identified in deep-sea waters [49,50], our study demonstrates that depth plays a previously unrecognized role in shaping viral diversity in these environments [3]. We found that, on a global scale, viral species diversity in the deep sea is significantly influenced by depth, with shallower waters harbouring a greater number of viral species than deeper layers. In contrast, the deepest waters contained a higher proportion of uncharacterized viruses, underscoring the largely unexplored nature of these ecosystems.

Furthermore, depth significantly influenced viral community composition. Beta diversity analysis of viral sequence reads revealed marked dissimilarities among viral communities, with depth-related differences accounting for a substantial portion of this variation (R²=0.16). The Bray–Curtis dissimilarity index, which measures differences between samples based on the relative abundance of each species and gives greater weight to more abundant taxa [51], was used to assess community composition. As suggested by previous studies [52], these dissimilarities may be driven by variations in the abundance of dominant taxa, as well as differences in the taxonomy and abundance of rare community members [52,53].

The viral community was dominated by the Caudoviricetes and *Malgrandaviricetes* classes, consistent with previous studies [11]. At 270–1,000 m, *Sinsheimervirus phiX174*, a representative of Malgrandaviricetes, was significantly abundant (padj=5.26e-14), reflecting the dominance of this class at mid-depths. In contrast, at 2,400–4,005 m, *M. mendera* was the most abundant (padj=1.51e-08), corresponding with the increased prevalence of Caudoviricetes at greater depths.

We observed that the use of different bioinformatics pipelines can generate putative viral sequences with distinct characteristics. For example, VirSorter2 v2.2.3 [32] identified a higher average number of viral sequences and produced larger average assembly sizes compared to VIBRANT v1.2.0 [33]. The performance of VirSorter2 v2.2.3 can be attributed to its advanced set of customized automatic classifiers and genome-informed databases, which have been demonstrated to enhance both the accuracy and scope of viral sequence detection [32]. The pipeline used in this work generated 7,574 putative viral sequences (contigs ≥5 kb), which is significantly higher than the 2,099 contigs reported by Yu *et al*. [12], who also focused on contigs ≥5 kb.

We determined that both the number of contigs and the size of the largest contigs were significantly affected by depth, with the highest average number of contigs observed at 270–1,000 m (137 on average). Additionally, a smaller proportion of viral contigs from 2,400 to 4,005 m was identified as known viruses in the NCBI RefSeq database, compared to those from 270 to 1,000 m. This difference may be due to the fact that viruses at greater depths are less studied and thus less represented in public databases.

Consistent with the sequence read profiles, species diversity based on viral sequence contigs was significantly influenced by depth (Wilcoxon test, *P*=8.6e-10), with a greater number of species and higher evenness observed at 270–1,000 m (*P*=3e-6). Furthermore, viral community composition was significantly structured by depth (*P*=0.001).

Viral sequence contigs were predominantly identified as dsDNA viruses, with higher abundance observed at 270–1,000 m. A substantial portion of these contigs was classified as viruses not represented in current reference databases, with unclassified Caudoviricetes and Uroviricota being the most frequently detected, consistent with previous findings [3,11]. Caudoviricetes, a class of tailed dsDNA phages, are among the most recovered dsDNA viruses from environmental samples [11]. However, it is important to note that current viral reference databases still capture only a limited fraction of viral diversity [11]. At the species level, unclassified viruses were most abundant at 2,400–4,005 m, with an average relative abundance of 31.9%, compared to 9.6% at 270–1,000 m. This scarcity of deep-sea viral sequences in public databases underscores our limited understanding of this ecosystem and highlights the significant scientific challenge it presents on a global scale.

Depth also seems to affect viral metabolism, with a higher abundance of AMGs observed at 270 to 1,000 m. The most prevalent AMGs at this depth were associated with cofactor and vitamin metabolism, nucleotide metabolism and glycan metabolism. Previous studies have reported that a large portion of AMGs in deep-sea viruses participate in carbohydrate metabolism, cofactor and vitamin metabolism and amino acid metabolism [11]. Enzymes such as DNA (cytosine-5)-methyltransferase (K00558, *DNMT*) have been identified in viromes from seamount sediment samples [11]. Viromes enriched for lytic viruses have been reported to exhibit higher relative abundances of enzymes involved in fatty acid synthesis (e.g. *gmhA*), which enhances viral progeny reproduction [11]. AMGs could potentially facilitate the decomposition and utilization of complex carbohydrates in hosts in the deep sea, thereby enhancing host adaptation to their environments [11].

Our comprehensive analysis of viral sequences of the global deep sea underscores the significant impact of depth on the diversity and composition of viral communities in this unique and still unexplored ecosystem. We observed that shallower depths host a greater number of viral species with more even distribution, while deeper depths exhibit a higher proportion of unclassified viral taxa. Depth not only influences microbial viral diversity but also significantly shapes viral community composition. We also highlight the dominance of Caudoviricetes in the two depth profiles analysed. Additionally, the use of different bioinformatics pipelines, such as VirSorter2, demonstrated varying efficiencies in identifying viral sequences, suggesting that methodological advances are crucial for enhancing our understanding of microbial viral diversity. The significant presence of AMGs at certain depths further suggests that viruses may play important roles in host metabolism and adaptation in the deep sea. Our findings underscore the importance of ongoing exploration and characterization of deep-sea viral ecosystems to fully understand their complexity and ecological significance.

## Supplementary material

10.1099/mic.0.001632 Uncited Supplementary Material 1.

10.1099/mic.0.001632 Uncited Supplementary Material 2.

## Abbreviations

AMGs: auxiliary metabolic genes
KEGG: Kyoto Encyclopedia of Genes and Genomes
LCA: last common ancestor
NMDS: non-metric multidimensional scaling
PERMANOVA: permutational multivariate analysis of variance
