# Supplementary material for: Depth-driven decline in viral diversity unveils potential novel viruses in global deep-sea ecosystems
Source: Microbiology (Reading). 2025 Dec 10;171(12):001632. doi: 10.1099/mic.0.001632 (PMC12694929; doi:10.1099/mic.0.001632)
Supplement: Uncited Supplementary Material 1. [file mic-171-01632-s001.pdf]

Depth-Driven Decline in Viral Diversity Unveils Novel Viruses in Global Deep-Sea Ecosystems

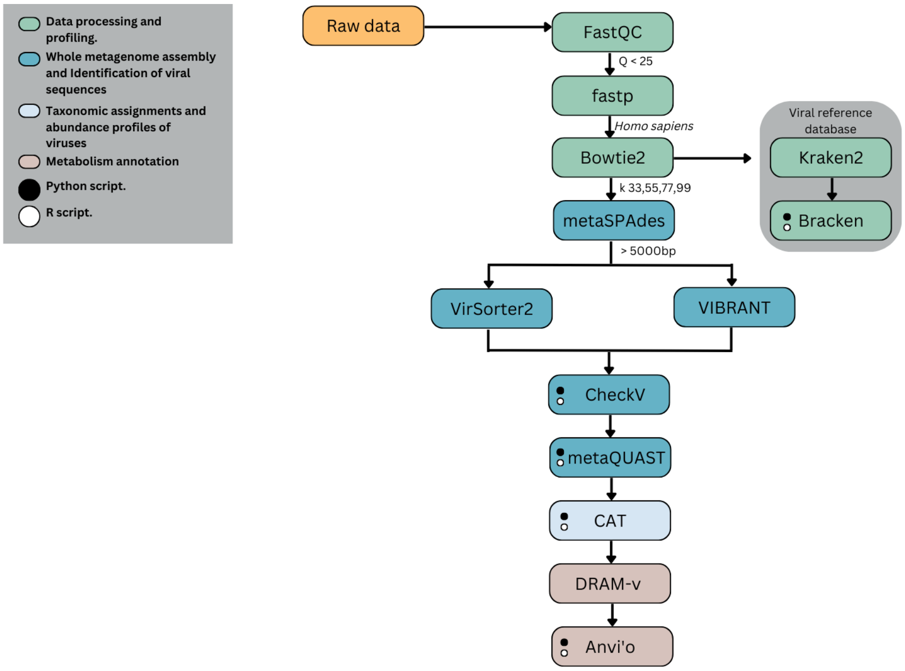

Fig. S1. Pipeline strategy proposed for characterizing the viromes of whole metagenomes from the deep-sea. Black dots represent steps where Python scripts were employed, while white dots represent steps where R scripts were used.

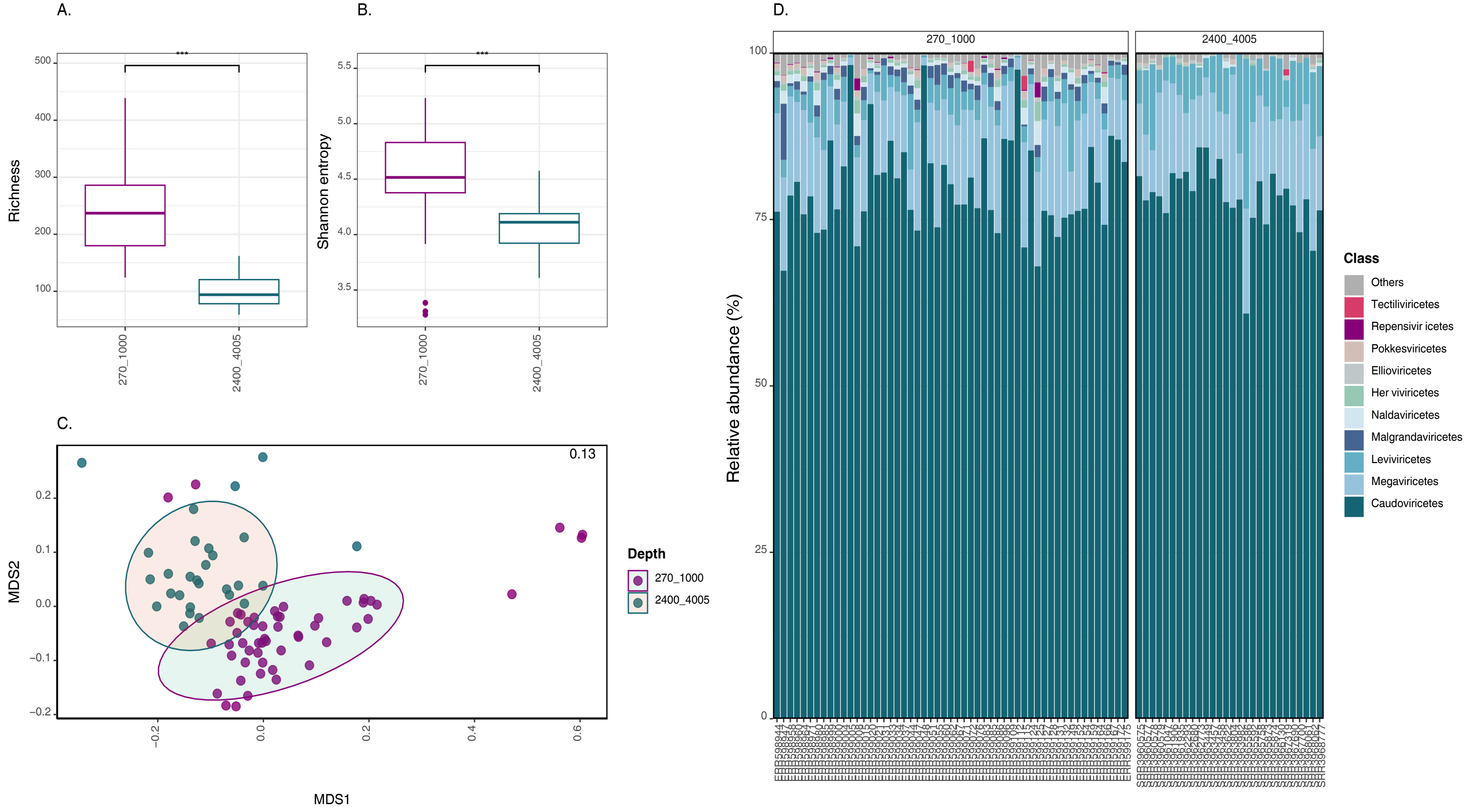

Fig. S2. Diversity on virus sequence reads. (a). Richness of viral communities. (b). Shannon entropy of viral communities. (c). Non-metric multidimensional scaling (NMDS) plot representing the viral community using Bray-Curtis dissimilarity. (d). Relative abundance of the main taxonomic groups at class level in deep-sea.

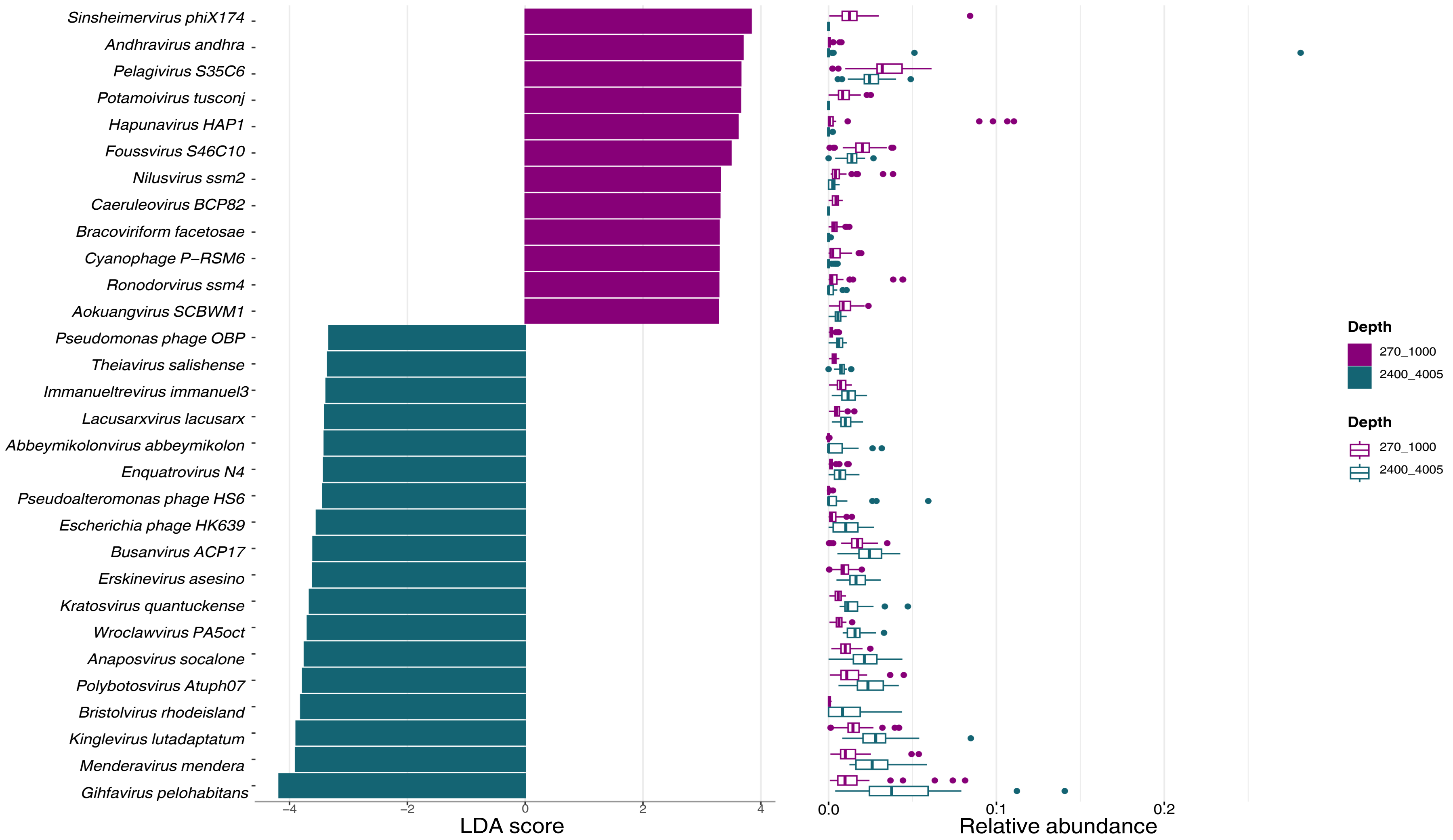

Fig. S3. Differentially abundant viral species across depth groups. The left panel presents Linear Discriminant Analysis (LDA) scores, identifying viral species enriched at different depths. Positive LDA scores (purple bars) indicate species enriched at 270–1000 m, while negative LDA scores (teal bars) indicate enrichment at 2400–4005 m. The right panel shows boxplots of relative abundance for each viral species, grouped by depth. Only species with significant differential abundance (p-adjusted < 0.01) are displayed.

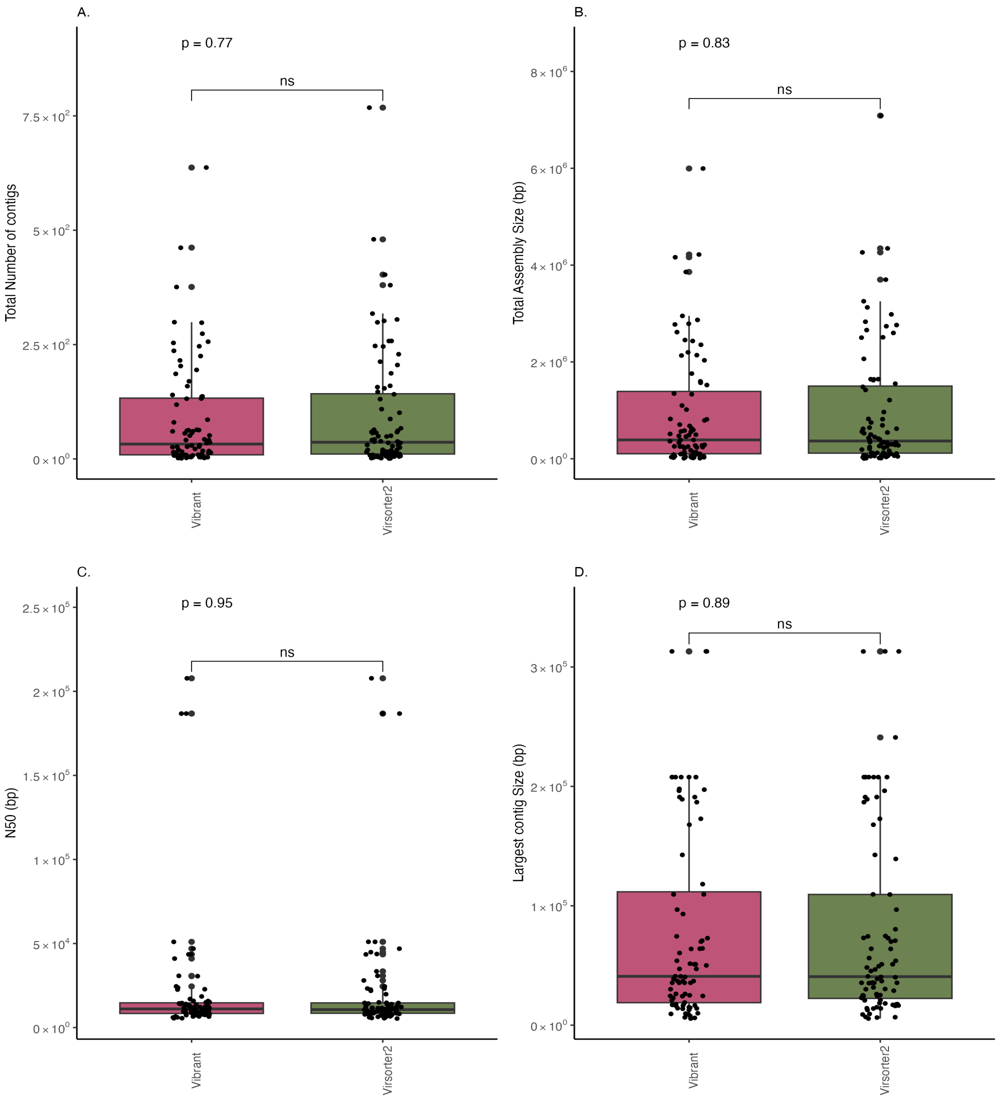

Fig. S4. Descriptive assembly metrics for virus assemblies using both strategies. (a) Total number of contigs produced. (b) Total assembly size in basepairs. (c) N50 of contig lengths. (d) Largest contig size in basepair

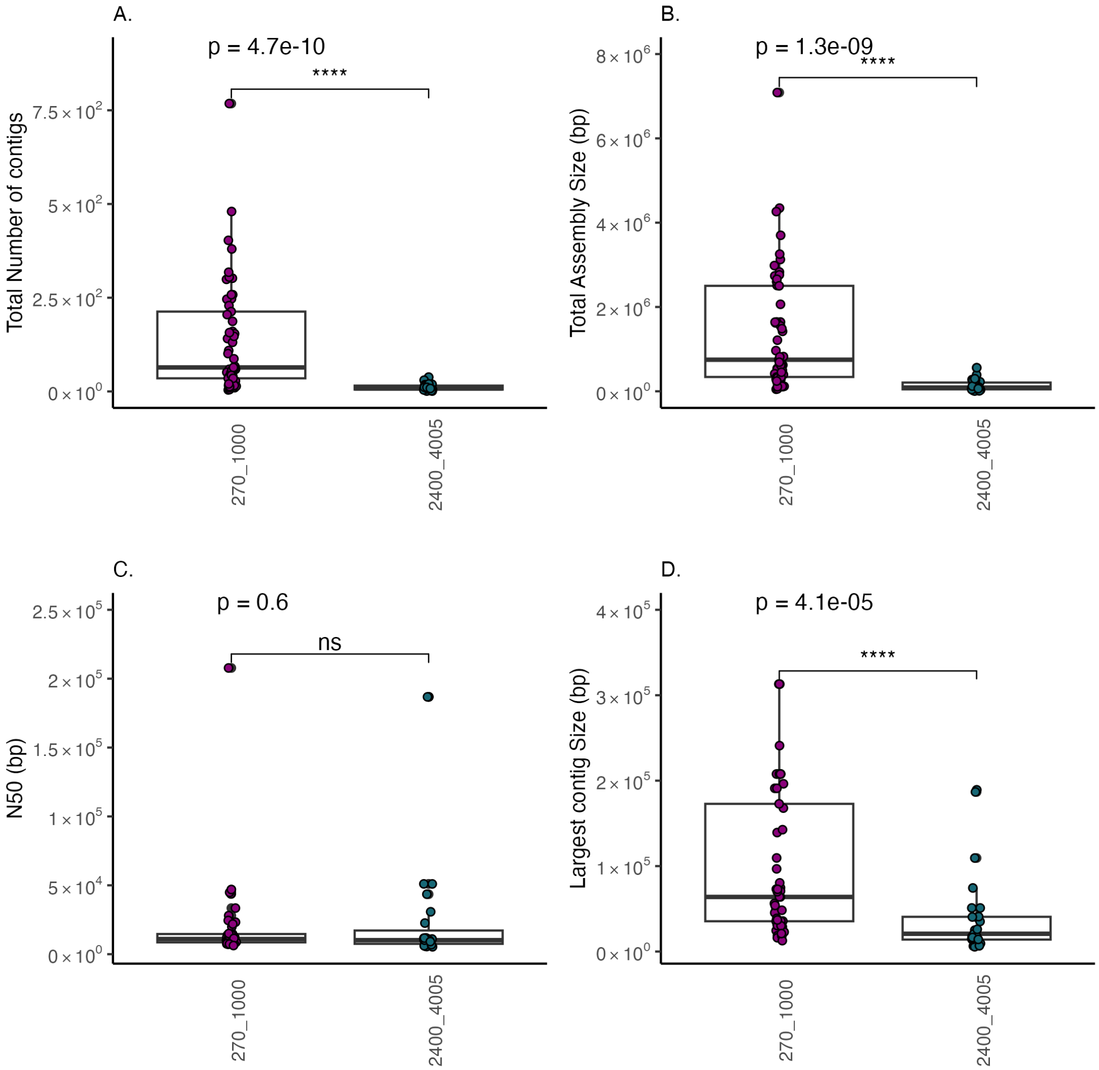

Fig. S5. Descriptive assembly metrics for virus assemblies using Virsorter2 recovery. (a) Total number of contigs produced. (b) Total assembly size in basepairs. (c) N50 of contig lengths. (d) Largest contig size in basepair

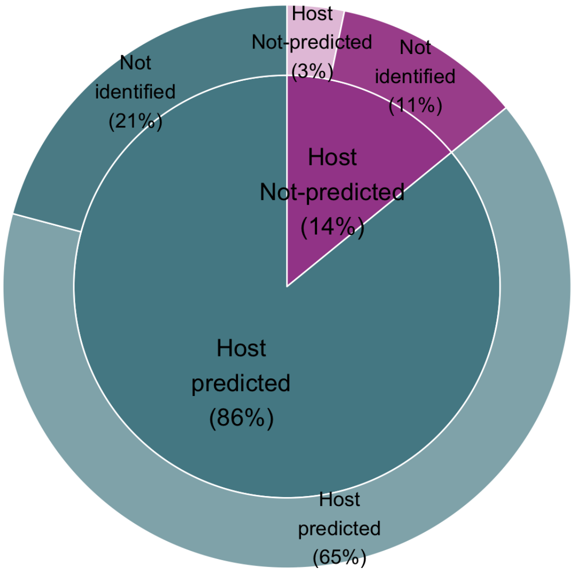

Fig. S6. Proportion of known and unknown viruses identified by both pipelines. The inner plot shows the proportions identified by VirSorter2, while the outer plot displays those identified by VIBRANT.

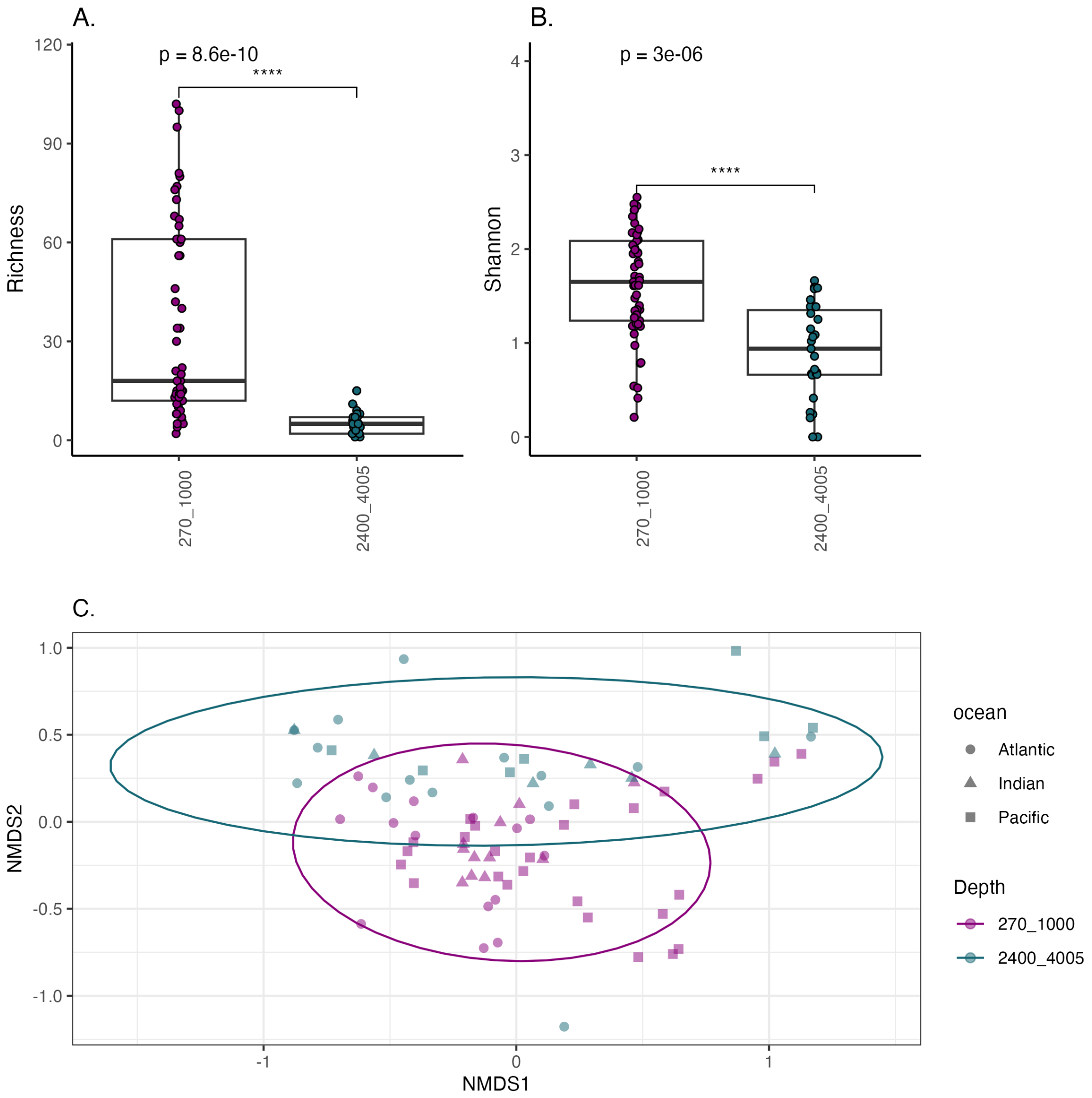

Fig. S7. Viral Contig Diversity (a). Richness of viral communities. (b). Eveness of viral communities. (c). Non-metric multidimensional scaling (NMDS) plot representing the viral community using Bray-Curtis dissimilarity.

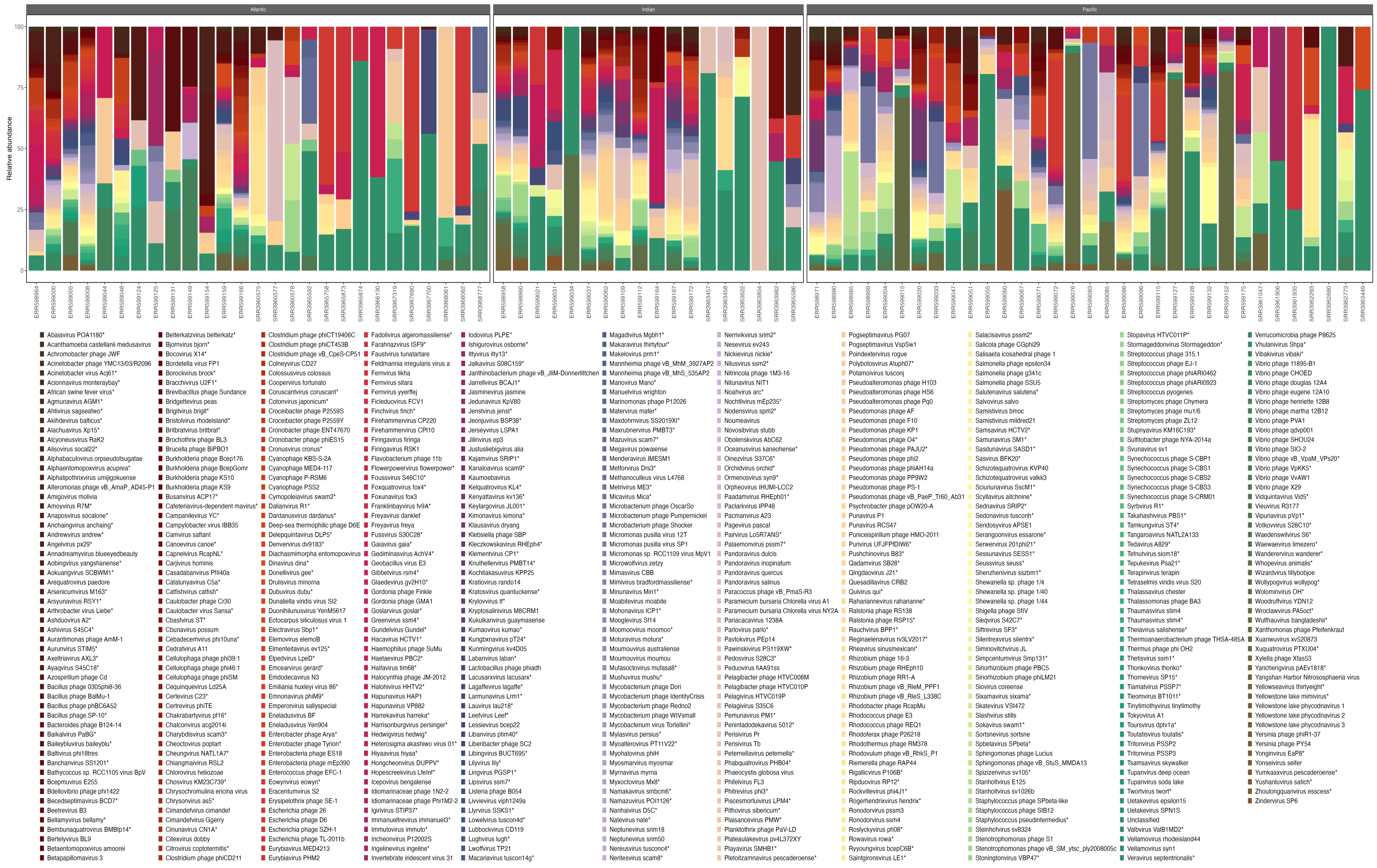

Fig. S8. Relative abundance at species in deep-sea, identified in viral sequence contigs.

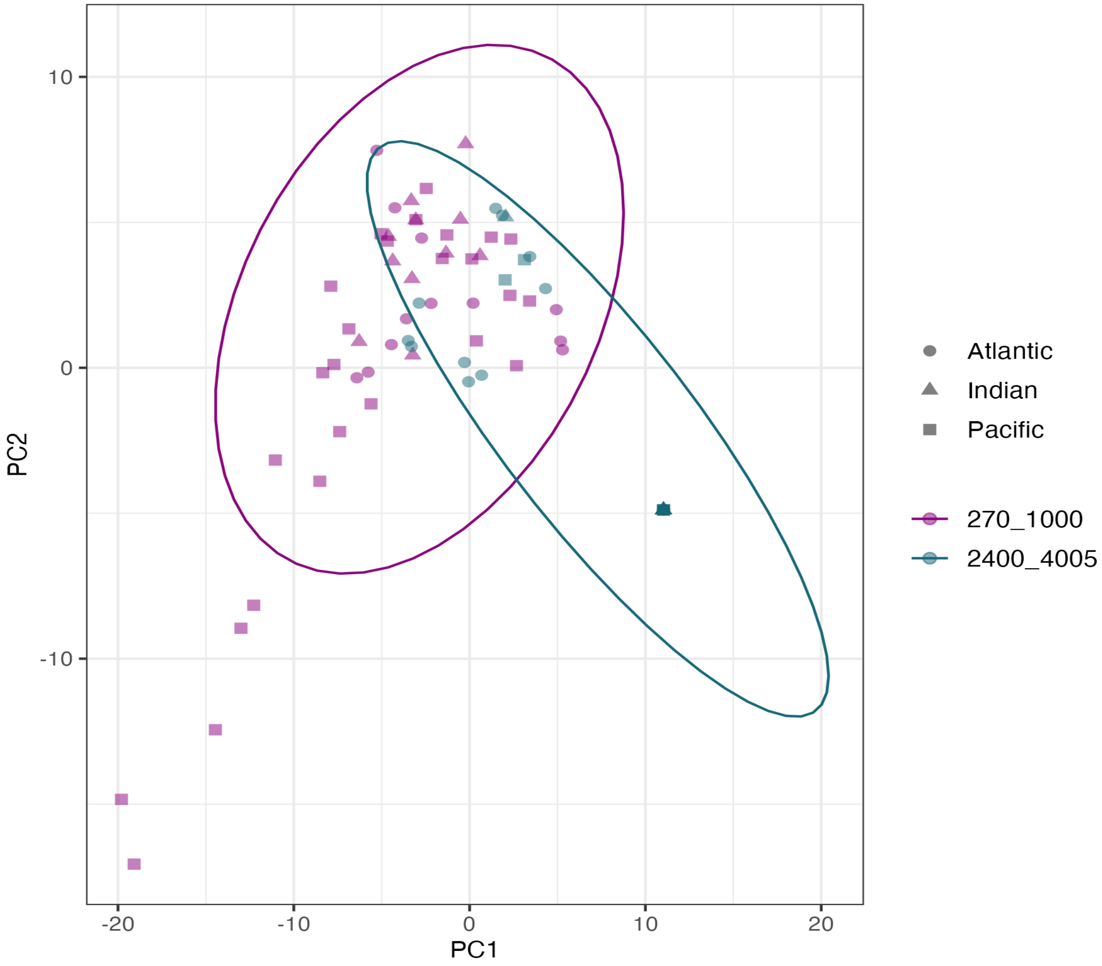

Fig. S9. Clustering of Auxiliary Metabolic Genes (AMGs) identified in viral sequence contigs.

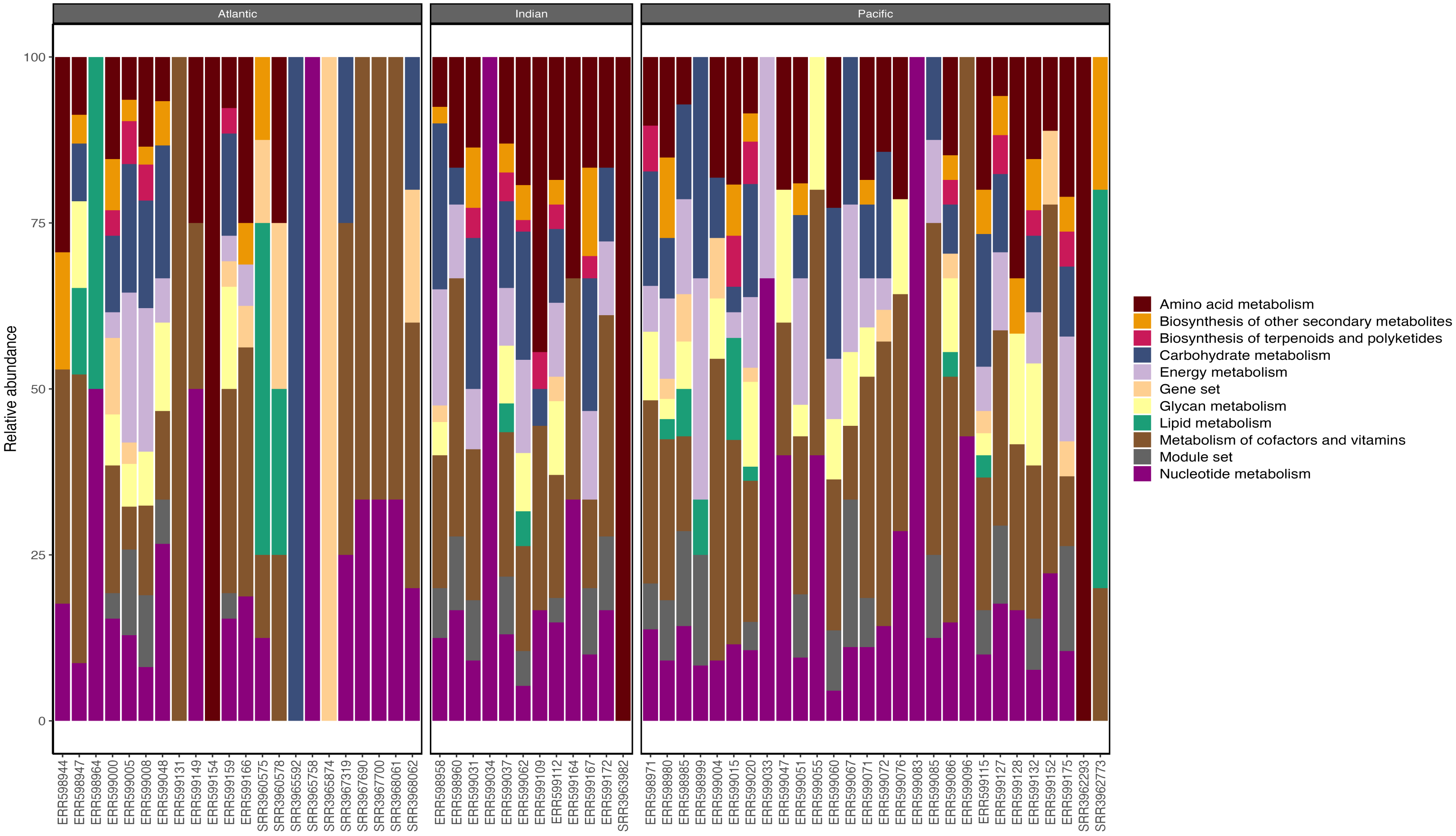

Fig. S10. Relative abundance of KEGG modules identified in viral sequence contigs.
